# Efficacy and safety of Puerarin injection on acute heart failure: A systematic review and meta-analysis

**DOI:** 10.3389/fcvm.2022.934598

**Published:** 2022-07-25

**Authors:** Zunjiang Li, Ye Fan, Chunxia Huang, Quanle Liu, Manhua Huang, Baijian Chen, Zhe Peng, Wei Zhu, Banghan Ding

**Affiliations:** ^1^The Second Affiliated Hospital of Guangzhou University of Chinese Medicine, Guangzhou, China; ^2^The Second Clinical College of Guangzhou University of Chinese Medicine, Guangzhou, China; ^3^Guangdong Provincial Hospital of Chinese Medicine, Guangzhou, China

**Keywords:** acute heart failure, Puerarin injection, meta-analysis, systematic review, traditional Chinese medicine

## Abstract

**Objective:**

This study aimed to assess the adjunctive efficacy and safety of Puerarin injection (PI) on acute heart failure (AHF) based on a systematic review and meta-analysis.

**Methods:**

Nine databases were searched from March 1990 to March 2022 to identify randomized controlled trials (RCTs) related to the adjunctive treatment of PI for AHF. The Cochrane collaboration tool was used to assess the risk of bias in the included studies. Meta-analysis and subgroup and sensitivity analyses were conducted by RevMan 5.3 software. The evidence’s certainty was evaluated by grading recommendations assessment, development, and evaluation (GRADE) methods.

**Results:**

A total of 8 studies were included with a total of 614 patients with AHF. The meta-analysis demonstrated that adjunctive treatment with PI on AHF was superior to conventional medicine alone. It increased the total effective rate (RR = 1.38; 95% CI, 1.22–1.55; *p* < 0.001) and improved left ventricular ejection fraction [SMD = 0.85; 95% CI (0.62, 1.09); *p* < 0.001]. Regarding safety, a total of 11.9% (23/194) adverse reactions were observed in the PI group and 9.8% (19/194) adverse reactions in the control group, and there were no significant differences in the incident rate of adverse events between both groups [RR = 1.16; 95% CI (0.66–2.05); *p* = 0.061]. The outcomes’ evidentiary quality was assessed as “moderate.”

**Conclusion:**

PI had an adjunctive effect on AHF combined with conventional medicine, and it seemed to be safe and more effective than the conventional medical treatment alone for improving the total clinical effective rate and left ventricular ejection fraction. But further well-designed RCTs are required to confirm the efficacy and safety of XBP in treating AHF due to the poor methodological quality of the included RCTs.

**Systematic Review Registration:**

[https://www.crd.york.ac.uk/PROSPERO/display_record.php?RecordID=327636], identifier [CRD42022327636].

## Introduction

Acute heart failure (AHF) is a clinical complex syndrome characterized by rapid deterioration and reduction in ventricular function necessitating hospitalization (1, 2). It has a prevalence of more than 23 million worldwide, associated with significant mortality, morbidity, and healthcare expenditures (3, 4). Significant drug advances have been developed and recommended in the treatment of patients with AHF in the past decades, including diuretic drugs, positive inotropes, vasodilators, neurohormonal antagonists, mechanical circulatory support, respiratory management, etc., [2021; (3, 5)], while none of the treatments tested to date have been definitively proven to improve AHF survival (6). Regarding patients with acutely decompensated HF or HF with preserved ejection fraction, approximately 50% of HF patients with preserved ejection fraction die within 5 years (5), and up to one in six patients with acute decompensation HF die during admission or within 30 days after discharge (4). Thus a new and an alternative drugs management of AHF is still challenging and of imperative need.

Puerarin (*7,4*′*-dihydroxy-8-C-glucosylisoflavone*) is the major bioactive ingredient of the root of *Radix Puerariae*, which was isolated in the late 1950s (7). Puerarin injection (PI) has been widely applied for the adjunctive management of coronary heart disease treatment and its main drug delivery method is intravenous injection (8). Clinical and experimental research proved that PI combined with conventional treatment could further improve the curative unstable angina pectoris (8, 9). PI could dilate coronary artery, increase coronary blood flow, decrease heart rate, inhibit platelet aggregation, and improve microcirculation (8, 10, 11). Literatures continuously reported clinical adjunctive efficacy and safety of PI, as well as their experimental effect and mechanism in animal models on AHF, but they still lacked relevant reviews summarizing the efficacy and safety of PI in the treatment of AHF in terms of the quality of methodology and evidence.

In the present study, we aimed to clarify the efficacy and safety of PI as an adjunctive treatment for acute heart failure (AHF) based on the available evidence in clinical practice. We mainly focused on clarifying whether PI had an adjunctive effect by combined use with conventional treatment and evaluating the safety of PI regarding its combined use.

## Data and methods

The effectiveness and safety of PI were critically assessed by a systematic review and meta-analysis according to the Preferred Reporting Items for Systematic Reviews and Meta-Analyses (PRISMA) (12).

### Database for search

A total of 5 English databases (the MEDLINE *via* PubMed, the Cochrane Library, EMBASE, the Web of Science and Ovid database) and 4 Chinese databases [China Science and Technology Journal Database (VIP), Chinese Biomedical Literature Database (CBM), Wan-fang Database, China National Knowledge Infrastructure (CNKI)] were searched for identifying studies from March 1990 to March 2022.

### Criteria for studies included

#### Type of participants (P)

Patients diagnosed with AHF in consistence with the AHF diagnostic criteria recognized at the time of publication of the study, regardless of age, gender, and course of the disease.

#### Type of interventions (I and C)

Control group: Conventional western medicine treatment, including diet and life regulation, diuretics, cardiotonic, oxygen inhalation, ECG monitoring, low-salt diet, restricted liquid intake etc. The treatment group was treated with PI in addition to the control group.

#### Type of outcome measures

Primary outcomes (O): ①Total clinical effective rate; ②left ventricular ejection fraction (LVEF); secondary outcomes: ①left ventricular end-diastolic dimension (LVEDD); ②isovolumic relaxation time (IVRT); ③peak A velocity of the mitral inflow; ④peak E velocity of the mitral inflow; ⑤stroke volume SV; safety outcome: adverse events.

#### Types of studies (S)

Randomized controlled trials (RCTs) of PI in the treatment of AHF, without limit on method and language.

### Exclusion criteria

①Repeated publications; ②case report; ③pure theoretical research; ④The data in the literature were wrong or incomplete.

### Searching strategy

The MeSH terms of PICOS were combined to search in [Title/Abstract] by developing our search strategies sequentially. A combination of P+I, P+I+C, P+I+C+O, and P+I+C+O+S was used to search for studies. If the number of searched studies were small, we would search as P+I. The artificially screened studies according to the included and excluded criteria and the searching strategy are detailed in Supplementary File 1.

### Data collection and analysis

#### Selection of studies and Kappa-coefficient analysis

After two review authors search out the articles, another two authors retrieved full text after screening the titles and abstracts, which meet with criteria of PICOS. Any discrepancies were handled by a discussion among all the authors. Then Kappa-coefficient analysis was performed regarding the level of agreement among the reviewers in article selection.

#### Data extraction and management

For data extraction, two reviewers independently identified the details for each study and presented them in a standardized form. The author’s name, published year, sample size, initial characteristics of patients, treatment detail, criteria for AHF diagnosis, outcomes and adverse reactions, etc., were extracted by two authors independently.

#### Evaluation of risk of bias

The quality evaluation was assessed by the risk of bias assessment tool recommended by Cochrane Handbook 5.1. Seven aspects were assessed by two review authors, including random sequence generation (selection bias), allocation concealment (selection bias), blinding of participants and personnel (performance bias), blinding of outcome assessment (detection bias), incomplete outcome data (attrition bias), selective reporting (reporting bias), and other sources of bias. The quality evaluation was judged as “high,” “low,” or “unclear” risk of bias. Any discrepancies were handled by consensus.

#### Data synthesis and analysis

The effect size was pooled using the Review Manager Software tool (RevMan, v.5.3; The Cochrane Collaboration). A fixed-effect model was chosen for the pool that had low heterogeneity, and a random-effects model was used where there was high heterogeneity. Mean deviation (MD) or Std mean difference (SMD) and 95% confidence intervals (CI) were utilized for continuous data, and relative risk (RR) with 95% CI were calculated for dichotomous data. Subgroup analysis and sensitivity analysis were also used to investigate potential sources of heterogeneity.

#### Sensitivity analysis

Sensitivity analysis was used to explore the significant heterogeneity that existed in studies, aiming to assess whether the conclusions were robust to the decision-making process. This study conducted a sensitivity analysis to observe whether the new effect-size results and heterogeneity changed significantly after removing single studies.

### Evidence confidence

The grading recommendations assessment, development, and evaluation (GRADE) technique (13) were used to assess the evidence’s certainty following the instructions of the website^1^. RCT evidence was initially classified as high quality, but it would be downgraded due to the risk of bias, inaccuracy, inconsistency, informality, and publication bias. The level of evidence was classified into four categories: “high,” “moderate,” “low,” and “very low.”

## Results

### Results of randomized controlled trials selection

**FIGURE 1:**
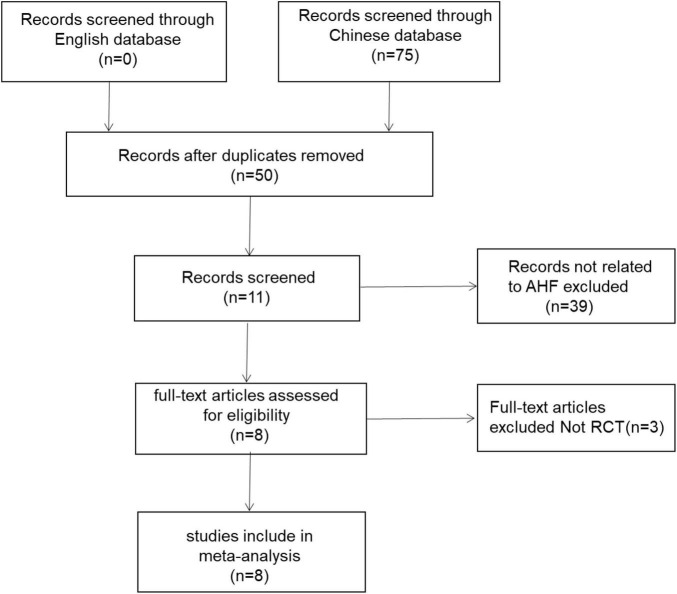
A PRISMA flow diagram of the literature screening and selection process.

A total of 75 related articles were initially detected. After 25 duplicate studies were eliminated, 50 RCTs were included for further screening. Then 39 studies were excluded without matching the inclusion requirements, and 3 non-RCT studies were eliminated after reviewing the article in detail. Finally, 8 studies (14–21) with a total of 614 patients with AHF were incorporated for systematic review and meta-analysis. Kappa-coefficient analysis suggested that the level of agreement among the two reviewers in article selection had a high degree of consistency (Kappa = 0.805, Supplementary Table 1). Figure 1 depicted the literature screening process and results.

### Characteristics of the included randomized controlled trials

All 8 included RCTs were conducted in China between 2012 and 2019, the sample size ranged from 58 to 100, and the treatment duration varied from 7 days to 14 days, except in one study (21) which had no report on duration. All research interventions were Puerarin injection (PI) in combination with conventional western treatment, and the drug delivery methods of PI were intravenous injection in all the studies. In terms of the usage and dose of PI, 3 studies diluted 500 mg of Puerarin with 500 ml 5% glucose (14, 16, 17), 2 studies diluted 500 mg Puerarin with 250 ml 5% glucose (18, 21), and 3 studies diluted 200–400 mg with Puerarin with 500 ml of 5% glucose (15, 19, 20). Only 3 studies (14, 15, 17) reported that the AHF diagnostic criteria was inconsistent with acute heart failure diagnosis and treatment guide (2010 version) published by the Cardiovascular Disease Branch of Chinese Medical Association (22). None of the studies reported follow-up results. The basic characteristics of included RCTs are detailed in Table 1.

**TABLE 1:** Characteristics of included RCTs investigating the adjunctive effect of Puerarin injection (PI) on acute heart failure.

Included study (author/year/ language)	Sample size (E/C)	Average age (E/C)	Duration	Interventions		Usage and dose	AHF diagnostic criteria	Adverse events	Outcome
				
				Experiment group	Control group				
Ma (15)	40/40	60.35 ± 6.55/60.40 ± 6.53	20 days	PI plus CWT+M	CWT+M	200–400 mg diluted with 5% glucose 500 ml	a	Nausea, hypotension, vomiting, headache	①⑥
Zheng et al. (16)	42/42	60.6 ± 10.2/54.3 ± 13.5	14 days	PI plus CWT+M	CWT+M	500 mg diluted with 5% glucose 500 ml	NR	Nausea, hypotension, vomiting, headache	①②③⑤⑥
Zhang (20)	34/34	56.3 ± 5.8/56.8 ± 5.3	14 days	PI plus CWT+M	CWT+M	200–400 mg diluted with 5% glucose 500 ml	NR	Nausea, hypotension, vomiting, headache	①②③⑥
Xu (14)	33/33	61.37 ± 5.62/63.35 ± 4.13	7 days	PI plus CWT+L	CWT+L	500 mg diluted with 5% glucose 500 ml	a	No adverse events	①②③⑤⑥
Li (17)	29/29	60.21 ± 3.05/60.13 ± 3.11	14 days	PI plus CWT+M	CWT+M	500 mg diluted with 5% glucose 500 ml	a	NR	①②
Wu (19)	34/34	67.28 ± 3.10/66.03 ± 3.87	7 days	PI plus CWT+ rhBNP	CWT+ rhBNP	200–400 mg diluted with 5% glucose 500 ml	NR	NR	②
Wang (18)	50/50	58.05 ± 1.25/57.15 ± 1.46	14 days	PI plus CWT+M	CWT+M	500 mg diluted with 5% glucose 250 ml	NR	NR	②
Xiong (21)	45/45	58.96 ± 8.15/58.87 ± 8.21	NR	PI plus CWT+M	CWT+M	500 mg diluted with 5% glucose 250 ml	NR	Slow heart rate, hypotension, headache	①②③④⑥

E/C, Experimental group/Control group; PI, Puerarin injection; CWT, conventional western treatment; M, Metoprolol; rhBNP, Recombined human; NR, Not report; ①: Total Effective Rate; ②LVEF: Left ventricular ejection fraction; ③LVEDD: Left ventricular end-diastolic dimension; ④SV: Stroke volume; ⑤NT-proBNP: N-terminal pro-B-type natriuretic peptide; ⑥Adverse events. a. Acute heart failure diagnosis and treatment guide (2010 version) published by Cardiovascular Disease Branch of Chinese Medical Association.

### Risk of bias assessment

One trial (16) was rated as low risk for using random number tables to generate sequences, while the other studies (14, 15, 17–21) provided no details about the method of random sequences generation. All the included studies published complete data, and no selective outcomes were reported, so the risk of bias was considered “low.” Beyond that, no studies mentioned the information about concealing of allocation, blinding of researchers, participants, and outcome evaluators, resulting in the risk of bias regarding performance, and detection was considered “unclear.” The risk of other bias was considered “low,” since no other obvious bias was observed in all RCTs. Table 2 shows the results of the risk of bias of the included RCTs.

**TABLE 2:** The results of risk of bias of included RCTs.

Study	Random sequence generation (selection bias)	Allocation concealment (selection bias)	Blinding of participants and personnel (performance bias)	Blinding of outcome assessment (detection bias)	Incomplete outcome data (attrition bias)	Selective reporting (reporting bias)	Other source of bias
Ma (15)	Unclear	Unclear	Unclear	Unclear	Low	Low	Low
Zheng et al. (16)	Low	Unclear	Unclear	Unclear	Low	Low	Low
Zhang (20)	Unclear	Unclear	Unclear	Unclear	Low	Low	Low
Xu (14)	Unclear	Unclear	Unclear	Unclear	Low	Low	Low
Li (17)	Unclear	Unclear	Unclear	Unclear	Low	Low	Low
Wu (19)	Unclear	Unclear	Unclear	Unclear	Low	Low	Low
Wang (18)	Unclear	Unclear	Unclear	Unclear	Low	Low	Low
Xiong (21)	Unclear	Unclear	Unclear	Unclear	Low	Low	Low

### Meta-analysis results

#### Primary outcome measures of total effective rate

**FIGURE 2:**
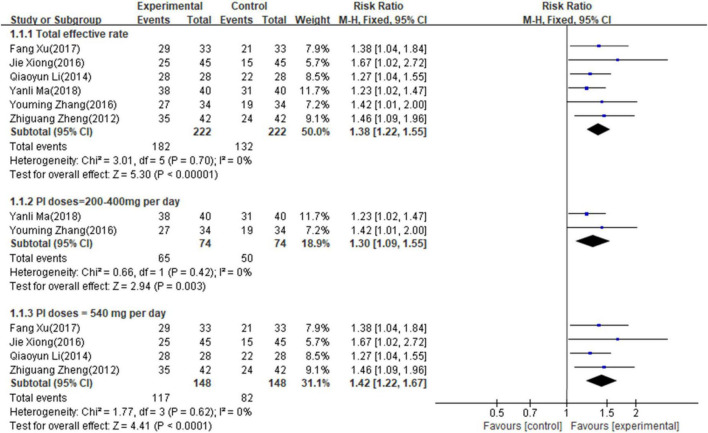
Forest plot of total effective rate (the total effective rate = effective rate + significant effective rate; invalid rate: patient’s heart function, physical signs, and clinical AHF symptoms have not been improved or even worsen; effective rate: patient’s heart function improved with 1 level, physical signs, and clinical AHF symptoms are relieved; significant effect rate: patient’s heart function improved with 2 level or more, the heart rate decreased to normal level, physical signs, and clinical AHF symptoms have disappeared).

Six studies (14–17, 20, 21) involving 444 patients reported the total effective rate. The fixed-effects model was used for meta-analysis as there existed little heterogeneity between the studies (*p* = 0.83, I^2^ = 0%). As shown in Figure 2, the results of the meta-analysis suggested that PI combined with conventional medical treatment increased the total effective rate by comparing with conventional medicine alone (RR = 1.38; 95% CI, 1.22–1.55; *p* < 0.001), indicating that PI had a favorable adjunctive effect on the total effective rate of AHF. Subgroup analyses according to PI doses showed that 200–400 mg/day (RR = 1.30; 95% CI, 1.09–1.55; *p* = 0.003) and 500 mg/day (RR = 1.42; 95% CI, 1.22–1.67; *p* < 0.001) of PI combined with conventional medicines treatments both increased the total effective rate compared with conventional medicine alone.

#### Primary outcome measures of left ventricular ejection fraction

**FIGURE 3:**
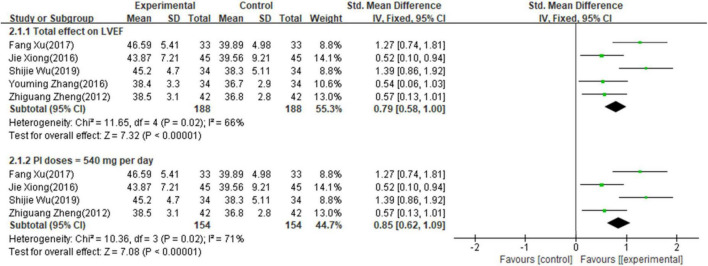
Forest plot of LVEF.

Seven studies involving 534 patients reported the results of LVEF. The random-effects model was used for meta-analysis as there existed high heterogeneity between studies (*p* < 0.001, I^2^ = 96%). The results of the meta-analysis indicated that combining PI with a conventional medical treatment significantly improved LVEF (RR = 1.07; 95% CI, 0.87–1.27; *p* < 0.001, Supplementary Figure 1). Sensitivity analyses were performed by excluding studies one by one. After removing the studies reported by “(17)” (17) and “(18)” (18), heterogeneity between studies was significantly reduced to 66%. As shown in Table 1, the sample size of the study “(17)” (17) and “(18)” (18) were the largest and smallest compared with other studies respectively, which might contribute to high heterogeneity. The results showed that the LVEF of patients with AHF was still significantly improved by the combined use of PI with conventional medical treatment (SMD = 0.79; 95% CI, 0.58–1.00; *p* < 0.001, Figure 3), and it indicated that combined use of PI was beneficial for LVEF in patients with AHF. Subgroup analyses showed that 500 mg/day of PI combined with conventional medicines treatments also improved the LVEF compared with conventional medicine alone (SMD = 0.85; 95% CI, 0.62–1.09; *p* < 0.001, Figure 3). As “(15)” (15) did not report the LVEF valve, there were no sufficient studies (≥2) for subgroup analyses on the dose of 200–400 mg/day.

#### Secondary outcome measures of other heart function indicators

**FIGURE 4:**
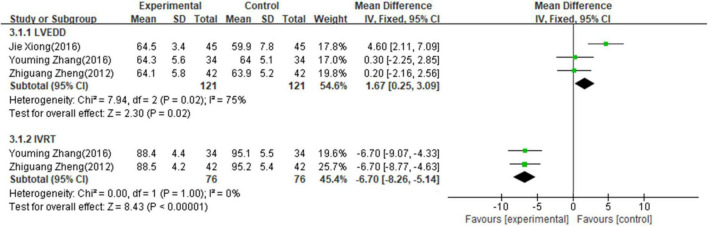
Forest plot of other heart function indicators.

Three studies (16, 20, 21) involving 242 patients reported the value of LVEDD and two studies (16, 20) involving 152 patients reported the value of IVRT. The fixed-effects model was used for meta-analysis on LVEDD (*p* = 0.02, I^2^ = 75%) and IVRT (*p* = 1.00, I^2^ = 0%) as there existed low to median heterogeneity between studies. As shown in Figure 4, the results of meta-analysis indicated that combining PI with conventional medicine treatment improved the heart function, including increased LVEDD (MD = 1.67; 95% CI, 0.25–3.09; *p* < 0.001, Figure 4) and decreased IVRT (MD = −6.70; 95% CI, −8.26 to −5.14; *p* < 0.001, Figure 4) when compared with conventional medicine alone.

#### Secondary outcome measures of left ventricular diastolic function

**FIGURE 5:**
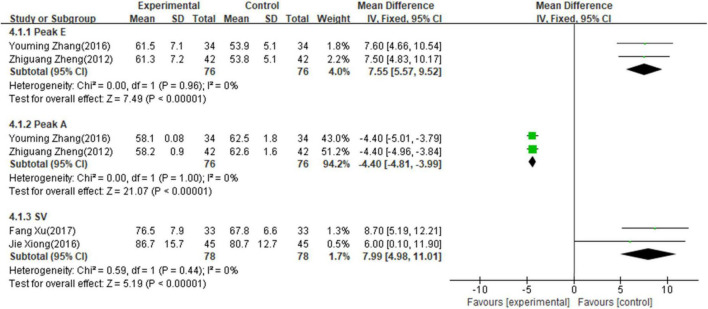
Forest plot of left ventricular diastolic function.

Two studies (16, 20) involving 152 patients reported the value of peak E, two studies (16, 20) involving 152 patients reported the value of peak A, and two studies (14, 21) involving 156 patients reported the value of SV. The fixed-effects model was used for meta-analysis on peak E (*p* = 0.96, I^2^ = 0%), Peak A (*p* = 1.00, I^2^ = 0%) and IVRT (*p* = 0.44, I^2^ = 0%) as there existed no heterogeneity between studies. As shown in Figure 5, the results of meta-analysis indicated that combining PI with conventional medicine treatment improved the left ventricular diastolic function, including increased peak E (MD = 7.55; 95% CI, 5.57–9.52; *p* < 0.001, Figure 5), decreased Peak A (MD = −4.40; 95% CI, −4.81 to −3.99; *p* < 0.001, Figure 5), and increased SV (MD = 7.99; 95% CI, 4.98–11.01; *p* < 0.001, Figure 5) when compared with conventional medicine alone.

### Safety of adverse events comparison

**FIGURE 6:**
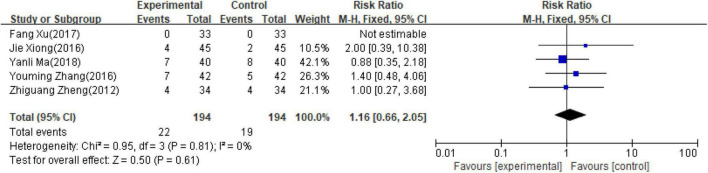
Forest plot of adverse effect.

Five studies (14–16, 20, 21) involving 388 patients reported adverse events. As detailed in Table 3, one study (14) reported no adverse reactions in both groups and one study (20) reported the total number of adverse effects without classification. Three studies (15, 16, 21) reported a detailed number of each kind of adverse reactions in both groups. In all, it reported a total of 11.9% (23/194) adverse reactions in the PI group and 9.8% (19/194) adverse reactions in the control group. All of the adverse reactions were modest, and no significant difference in the incident rate of adverse events was observed in both groups (RR = 1.16; 95% CI, 0.66–2.05; *p* = 0.061, Figure 6), indicating that adjunctive use of PI was safe as a conventional medical treatment.

**TABLE 3:** The incidence rate of adverse effect.

Adverse effect	Studies	Total number of adverse effects	
		
		Experiment group	Control group
Nausea	(15, 16)	4	5
Hypotension	(15, 16, 21)	6	3
Vomiting	(15, 16)	4	4
Headache	(15, 16, 21)	3	1
Slow heart rate	(21)	2	2
No detailed classification	(20)	4	4
No adverse effect	(14)	0	0
Total events		23/194	19/194
Incident rate		11.9%	9.8%

### Results of publication bias assess

**FIGURE 7:**
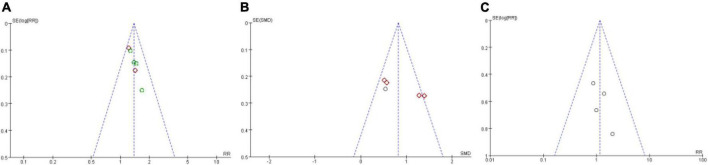
Funnel plot of publication bias assess. **(A)** Publication bias assess on total effective rate; **(B)** publication bias assess on LVEF; **(C)** Publication bias assess on adverse effect.

We assessed publication bias on the results of total effective rate, LVEF, and adverse effect, as other results had less than three studies included. We detected that there is no publication bias on the results of total effective rate, LVEF, and adverse effect (Figure 7). But because of lacking access to the information on the clinical trial registry or study protocol, it could not rule out the potential of selectively reporting existing results. The published bias result of other results are provided in Supplementary File 2.

### The quality of the evidence

The certainty of evidence on meta outcomes was assessed by the grading recommendations assessment, development, and evaluation (GRADE) methods, and it showed that evidentiary quality of meta results varied from “very low” to “moderate.” The rationale for the downgrade was mainly due to small sample sizes and unclear risk of bias in the selected studies, as shown in Table 4.

**TABLE 4:** The summary findings by the grading recommendations assessment, development, and evaluation (GRADE) methods.

Certainty assessment							Summary of findings			Comments
	
Participants (studies) follow-up	Risk of bias	Inconsistency	Indirectness	Imprecision	Publication bias	Overall certainty of evidence	Events		Anticipated absolute effects or relative effect (95% CI)	
							
							Control	Experiment		
Total Effective Rate 444 (6 RCTs)	Serious^a^	Not serious	Not serious	Not serious	None	⊕⊕⊕○ Moderate	132/222 (59.5%)	182/222 (82.0%)	RR 1.38 (1.22–1.55)	Risk of bias (-1^a^)
LVEF 376 (5 RCTs)	Serious^a^	Not serious	Not serious	Not serious	None	⊕⊕⊕○ Moderate	188	188	SMD 0.79 higher (0.58–1.00)	Risk of bias (-1^a^)
LVDD 242 (3 RCTs)	Serious^a^	Serious^b^	Serious^c^	Not serious	None	⊕⊕○○ Low	121	121	MD 1.67 higher (0.25–3.09)	Risk of bias (-1^a^) Inconsistency (-1^b^) Indirectness (-1^c^)
IVRT 152 (2 RCTs)	Serious^a^	Serious^b^	Not serious	Not serious^e^	Yes^d^	⊕○○○ Very low	76	76	MD 6.7 lower (8.28 lower to 5.14 lower)	Risk of bias (-1^a^) Inconsistency (-1^b^) Imprecision (-1^e^) Publication bias (-1^d^)
Peak A 152 (2 RCTs)	Serious^a^	Serious^b^	Not serious	Not serious^e^	Yes^d^	⊕○○○ Very low	76	76	MD 4.4 lower (4.81 lower to 3.99 lower)	Risk of bias (-1^a^) Inconsistency (-1^b^) Imprecision (-1^e^) Publication bias (-1^d^)
SV 156 (2 RCTs)	Serious^a^	Serious^b^	Not serious	Not serious^e^	Yes^d^	⊕○○○ Very low	78	78	MD 7.99 higher (4.98 to 11.01)	Risk of bias (-1^a^) Inconsistency (-1^b^) Imprecision (-1^e^) Publication bias (-1^d^)
Adverse Events 388 (5 RCTs)	Serious^a^	Not serious	Not serious	Not serious	None	⊕⊕⊕○ Moderate	19/194 (9.8%)	23/194 (11.9%)	RR 1.16 (0.66 to 2.05)	Risk of bias (-1^a^)

CI, confidence interval; MD, mean difference; RR, risk ratio; SMD, Standardized mean difference. (a.) There exists unclear risk of bias as showed in Table 2; (b.) The sample size was too small; c. The direction of the effect is different as 50 < I2 ≤ 75%; d. all plausible residual confounding would reduce the demonstrated effect; e. the number of studies is small. ⊕ The evidence’s confidence upgrade 1 level; ○ the evidence’s confidence downgrade 1 level.

## Discussion

Traditional Chinese medicine (TCM) is proved to have an adjunctive effect in treating diseases when combined with the use of western medicine in improving the symptoms and quality of life of patients (23). Due to the side effects of western medicine therapy, TCM integration with routine western or conventional medical interventions plays a significant adjunctive role in enhancing the therapeutical effect and reducing the occurrence of adverse effects (24), thus the unique advantages of TCM have received increasing attention, but it still lacks systematic overviews to summarize the effectiveness of TCM based on the existing clinical evidence. Our systematic review and meta-analysis contained eight RCTs and revealed that PI combination therapy had an adjunctive effect in the treatment of patients with AHF, it could better increase the total effective rate, improved the heart function, and be safe for adjunctive use in treating AHF.

### The adjunctive effect of Puerarin injection in treating acute heart failure

Our study also suggested that PI treatment in conjunction with conventional medical treatment was superior in increasing the total effective rate, improving heart function of the valve of LVEF, LVEDD, and IVRT, and improving the left ventricular diastolic function of the valve of Peak A, Peak E and stroke volume, in terms of comparing with conventional medical treatment alone. In addition, subgroup analysis was performed on the primary outcomes of total effective rate and LVEF. Interestingly, the results showed that adjunctive use of PI could both improve the LVEF value and increase the total effective rate regardless of the dose (200–400 mg/day or 500 mg/day). As previously reported, PI showed a satisfied clinical efficacy in the treatment of cardiovascular diseases that PI was more effective than using conventional western medical alone in the treatment of unstable angina pectoris (9). It is also more effective and relatively safe in the clinic for treating acute ischemic stroke and diabetic peripheral neuropathy (25, 26).

When we further explored the association between PI and favorable results in patients with AHF, it was proposed that PI had the effect of alleviating impaired heart function and inhibiting the levels of myocardial injury and inflammatory markers (27), as it was found that inflammation and heart function impaired in AHF resulted in neutral effects or worsening of clinical outcomes (28, 29). In addition, patients with AHF presented with similar congestion symptoms, which could lead to HF decompensation, which occurred owing to both fluid accumulation and redistribution, and further progress in the deterioration of AHF, thus decongestive therapy and diuretic drugs were recommended for AHF (1). PI was found that it could expand the coronary artery to promote coronary blood flow (10) and improve microcirculation to alleviate congestion symptoms (11), which might be the mechanism that PI could alleviate the AHF symptoms. Furthermore, clinical trials pointed out that a higher heart rate was a strong predictor of 1-year mortality of AHF, and reductions in coronary blood flow and myocardial oxygen consumption may be beneficial for AHF treatment (30, 31). Song et al reported that PI had the effect of decreasing heart rate and reducing myocardial oxygen consumption (32), which may also be the potential mechanism that PI had favorable results in patients with AHF.

### The safe of Puerarin injection in conjunction with conventional medicine in treating acute heart failure

Regarding clinical safety, a total of 9.8% (19/194) adverse reactions occurred in the control groups while 11.9% (23/194) in the PI group, including nausea, hypotension, vomiting, headache, and slow heart rate. As 5 (62.5%) studies (14–16, 20, 21) reported the adverse effects and moderate evidence for safety assessment, we preliminary put forward the argument that combination therapy of PI was safe in treating AHF. But since the record for risk of bias assessment of included RCTs was “unclear,” it implied that there is still a need for further eligible and critical clinical trials to validate the safety of PI.

### The assessment of bias risk and evidence’s confidence on the meta-results

The findings of meta-results were consistent with previously published research (33). To assess the credible clinical evidence of our results, evaluation of bias risk and evidence’s confidence were performed. It showed that all the included studies lack details in selection bias, blinding performance, and blinding outcome assessment (Table 2), which may result in the overstated effect of outcomes and reported bias in selected results. In addition, GRADE evaluation indicated that the confidence of the evidence was graded, which varied from very low to moderate quality for evidences (Table 4), and risk of bias, inconsistency, imprecision, and publication bias were mainly responsible for the downgrading of evidence because of the quality of included RCTs, thus larger RCTs with improved methodological quality in future are expected to further update the results of this systematic review.

### Implications of prospective research and limitations of the present study

The adjunctive efficacy and safety of PI regarding curative effect among patients with AHF were for the first time systematically reviewed and evaluated in this study. At present, AHF treatment still lacks specific and effective medicine, leading to a relatively high recurrence rate, hospitalization rate, and mortality rate. We found that integrated use with PI could improve heart function, increased total effective rate, and was safe as the conventional western medication, which could be chosen by physicians when patients with AHF faced with unexpected treatment effects. The methodology of the present study was designed to a high standard according to the methodological quality of systematic reviews-2 (AMSTAR 2) by identifying relevant literature comprehensively, developing evaluation plans, and strict implementation, which could improve the accuracy and clinical applicability of the results of this study (34).

Although the results were encouraging, restrictions were unavoidably present in this study. Due to the small number and low to moderate quality of included studies, strictly designed trials according to the Consolidated Standards of Reporting Trials (CONSORT) statement also need to be further performed to verify the efficacy of PI as an adjunctive therapy for AHF. Duration included 7 days and 14 days, and the dose of PI included 500 mg/day and 200–400 mg/day, but we only did perform subgroup analysis of dose on the total effective rate due to small number of studies. Besides, the control group involves different conventional medical treatments, which potentially led to heterogeneity between the studies. Although there was no restriction on language when screening literature, the final included studies were all performed in China, which may lead to potential selection bias in the research. In addition, a few have data available for each outcome, for instance, the number of studies included in the meta-analysis of LVEDD, IVRT, Peak A, Peak E, and SV was 2-3/8 (25–37.5%), which limited the credibility of the above results. Thus, much more caution should be taken about the results until further trials in different populations and high-quality designed studies were performed to strengthen and update the results of the present meta-results.

## Conclusion

In conclusion, PI plus CMT may be more beneficial than CMT alone for increasing the total effective rate, improve the heart function and left ventricular diastolic function. Also, it may be safe to combine PI with CMT in treating AHF. Regarding the very low to moderate evidence on the quality of meta-results, we should proceed with caution. Multi-center randomized controlled and double-blind trials are required with large sample sizes, rigorous design, and long follow-up period to confirm the efficacy and safety of PI in the future.

## Data availability statement

The original contributions presented in this study are included in the article/Supplementary Material, further inquiries can be directed to the corresponding author/s.

## Author contributions

ZL and YF provided conceptualization, methodology, investigation, and writing—original draft. CH helped provide methodology, investigation, and formal analysis. QL and BC helped provide investigation, validation, data collection, and visualization. MH and ZP helped provide data collection and validation. BD and WZ provided conceptualization, funding acquisition, supervision, writing—review and editing, and project administration. All authors contributed to the article and approved the submitted version.

## Conflict of interest

The authors declare that the research was conducted in the absence of any commercial or financial relationships that could be construed as a potential conflict of interest.

## Publisher’s note

All claims expressed in this article are solely those of the authors and do not necessarily represent those of their affiliated organizations, or those of the publisher, the editors and the reviewers. Any product that may be evaluated in this article, or claim that may be made by its manufacturer, is not guaranteed or endorsed by the publisher.

## Footnotes

1
https://www.gradepro.org/
