# Supplementary material for: Efficacy and safety of Puerarin injection on acute heart failure: A systematic review and meta-analysis
Source: Front Cardiovasc Med. 2022 Jul 25;9:934598. doi: 10.3389/fcvm.2022.934598 (PMC9357890; doi:10.3389/fcvm.2022.934598)
Supplement: Supplementary file 1 [file Data_Sheet_1.docx]

**Search strategy and search results for the bibliographic databases.**

1. Pubmed database

| Iterm | MeSH | Study number |
| --- | --- | --- |
| #1 | Puerarin Injection | 33 |
| #2 | Acute heart failure | 50836 |
| #3 | #1&#2 | 0 |

1. OVID database

| Iterm | MeSH | Study number |
| --- | --- | --- |
| #1 | Puerarin Injection | 33 |
| #2 | Acute heart failure | 7002 |
| #3 | #1&#2 | 0 |

1. Embase database

| Iterm | MeSH | Study number |
| --- | --- | --- |
| #1 | Puerarin Injection | 51 |
| #2 | Acute heart failure | 32434 |
| #3 | #1&#2 | 0 |

1. Web of Science

| Iterm | MeSH | Study number |
| --- | --- | --- |
| #1 | Puerarin Injection | 17 |
| #2 | Acute heart failure | 11685 |
| #3 | #1&#2 | 0 |

1. Cochrane library

| Iterm | MeSH | Study number |
| --- | --- | --- |
| #1 | Puerarin Injection | 48 |
| #2 | Acute heart failure | 9607 |
| #3 | #1&#2 | 0 |

1. China National Knowledge Infrastructure (CNKI)) database

| Iterm | MeSH | Study number |
| --- | --- | --- |
| #1 | Puerarin Injection | 60 |
| #2 | Acute heart failure | 4176 |
| #3 | #1&#2 | 9 |

1. Chinese Biomedical Literature Database (CBM) database

| Iterm | MeSH | Study number |
| --- | --- | --- |
| #1 | Puerarin Injection | 21 |
| #2 | Acute heart failure | 2165 |
| #3 | #1&#2 | 8 |

1. Wan-fang Database

| Iterm | MeSH | Study number |
| --- | --- | --- |
| #1 | Puerarin Injection | 2955 |
| #2 | Acute heart failure | 23084 |
| #3 | #1&#2 | 15 |

1. China Science and Technology Journal Database (VIP)

| Iterm | MeSH | Study number |
| --- | --- | --- |
| #1 | Puerarin Injection | 1422 |
| #2 | Acute heart failure | 4866 |
| #3 | #1&#2 | 8 |
