# Supplementary material for: Efficacy and safety of Puerarin injection on acute heart failure: A systematic review and meta-analysis
Source: Front Cardiovasc Med. 2022 Jul 25;9:934598. doi: 10.3389/fcvm.2022.934598 (PMC9357890; doi:10.3389/fcvm.2022.934598)
Supplement: Supplementary file 2 [file Data_Sheet_2.docx]

**Results of Publication Bias Assess**

As showed in the following figures, we assessed publication bias on the results of other results, including Left ventricular end diastolic Dimension (LVEDD), Isovolumic relaxation time (IVRT), Peak A velocity of the mitral inflow, Peak E velocity of the mitral inflow and Stroke volume SV. All the results showed that there had no publication bias on these indicators.


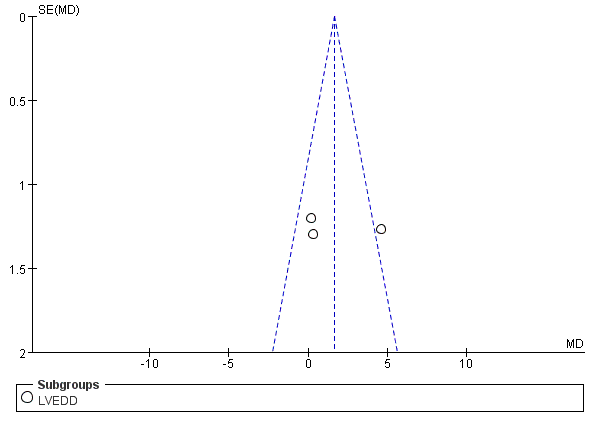


Funnel plot of publication bias assess on Left ventricular end diastolic Dimension (LVEDD)


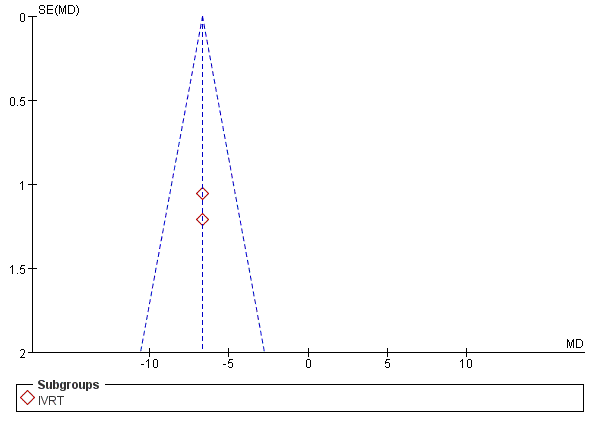


Funnel plot of publication bias assess on Isovolumic relaxation time (IVRT)


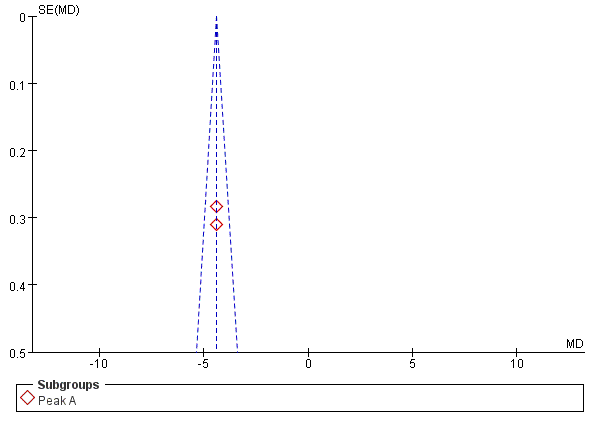


Funnel plot of publication bias assess on Peak A velocity of the mitral inflow


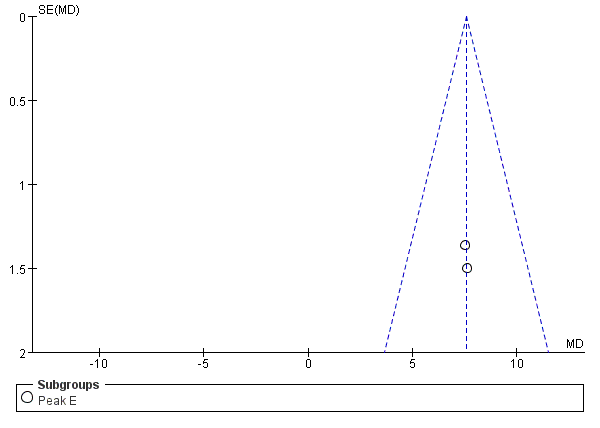


Funnel plot of publication bias assess on Peak E velocity of the mitral inflow


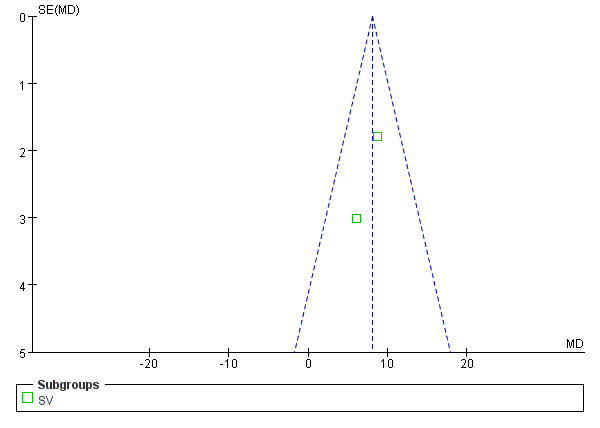


Funnel plot of publication bias assess on Stroke volume SV

**Definition of "Total Effective Rate"**

The definition of "total effective rate" were detailed described as the following criterion for the judgment of the effect(Ling, 2010):

Invalid rate: the rate of patient's heart function, physical signs, and clinical AHF symptoms have not been improved or even worsen;

Effective rate: the rate of patient's heart function improved with 1 level, physical signs, and clinical AHF symptoms are relieved;

Significant effective rate: the rate of patient's heart function improved with 2 level or more, the heart rate decreased to normal level, physical signs, and clinical AHF symptoms have disappeared.

The total effective rate=. Effective rate + Significant effective rate.

Heart function level are divided according to New York Heart Association Classification (Zhang et al., 2018):

NYHA Class Symptoms

I No limitation of physical activity. Ordinary physical activity does not cause undue fatigue, palpitation, dyspnea (shortness of breath).

II Slight limitation of physical activity. Comfortable at rest. Ordinary physical activity results in fatigue, palpitation, dyspnea.

III Marked limitation of physical activity. Comfortable at rest. Less than ordinary activity causes fatigue, palpitation, or dyspnea.

IV Unable to carry on any physical activity without discomfort. Symptoms of heart failure at rest. If any physical activity is undertaken, discomfort increases.

Significant effective rate includes the rate of patient's heart function improved with 2 level or more, it means that the patient’s heart function recovery from IV class to II class or even I class, from III to I class. Effective rate includes the rate of patient's heart function improved with 1 level, it means that the patient’s heart function recovery from IV class to III class, from III to II class or from II class to I class.

**References**

Ling, H. (2010). Acute heart failure diagnosis and treatment guide. Chinese Journal of Cardiology*.* 9(07), 557

Zhang, R., Ma, S., Shanahan, L., Munroe, J., Horn, S., and Speedie, S. (2018). Discovering and identifying New York heart association classification from electronic health records. BMC Med Inform Decis Mak*.* 18(Suppl 2), 48. doi: 10.1186/s12911-018-0625-7
