# Supplementary figures and images for: Efficacy and safety of Puerarin injection on acute heart failure: A systematic review and meta-analysis
Source: Front Cardiovasc Med. 2022 Jul 25;9:934598. doi: 10.3389/fcvm.2022.934598 (PMC9357890; doi:10.3389/fcvm.2022.934598)

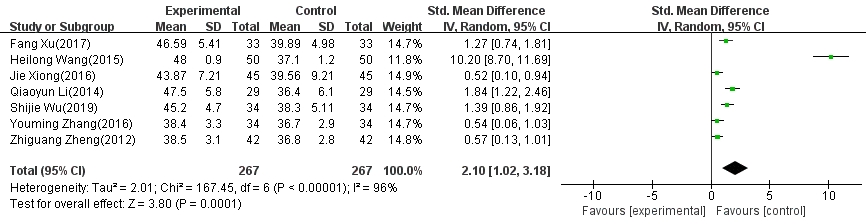

Supplement: Supplementary file 3 [file Image_1.jpeg]
